# Structural Study of a La(III) Complex of a 1,2,3-Triazole Ligand with Antioxidant Activity

**DOI:** 10.3390/antiox12101872

**Published:** 2023-10-17

**Authors:** Mauricio Alcolea Palafox, Nataliya P. Belskaya, Lozan T. Todorov, Irena P. Kostova

**Affiliations:** 1 Department of Physical Chemistry, Faculty of Chemical Sciences, Complutense University, 28040 Madrid, Spain; 2 Department of Technology for Organic Synthesis, Ural Federal University, 19 Mira Str., Yekaterinburg 620012, Russia; n.p.belskaya@urfu.ru; 3 Department of Chemistry, Faculty of Pharmacy, Medical University—Sofia, 2 Dunav Str., 1000 Sofia, Bulgaria; i.kostova@pharmfac.mu-sofia.bg

**Keywords:** 1,2,3-triazoles, antioxidant, lanthanum, vibrational analysis, scaling

## Abstract

The 1,2,3-triazole derivative 2-(4-chlorophenyl)-5-(pyrrolidin-1-yl)-2*H*-1,2,3-triazole-4-carboxylic acid with potential anticancer activity was used as a ligand in complex formation with the lanthanum(III) ion. The molecular structure and vibrational spectra of the complex were optimized at three DFT levels, and the scaled IR and Raman spectra were compared to the experimental ones. Several scaling procedures were used. Through a detailed analysis, the structure predicted for the newly synthetized La(III) complex was confirmed by the good accordance of the calculated/experimental IR and Raman spectra. The best DFT method appeared to be M06-2X with the Lanl2mb basis set, followed closely by Lanl2dz. The effect of the lanthanide atom on the molecular structure and atomic charge distribution of the triazole ring was evaluated. The potential free radical scavenging activity of both the ligand and the complex was investigated in several radical-generating model systems. The potential mechanisms of antioxidant action (hydrogen atom transfer (HAT) and single-electron transfer (SET)) were elucidated.

## 1. Introduction

**Figure 1:**
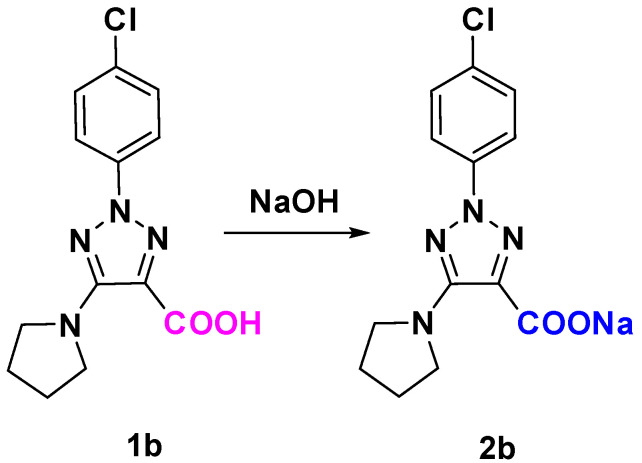
Structure of the 2-(4-chlorophenyl)-5-(pyrrolidin-1-yl)-2*H*-1,2,3-triazole-4-carboxylic acid (**1b**) and its sodium salt (**2b**).

Transition metals and their various organometallic and coordination compounds are widely studied and extensively used in medical practice [1,2]. Among them, rare earth elements have quickly gained prominence in cancer diagnosis and therapy, owing to their versatile chemical and magnetic properties due to their 4f electronic configuration and low toxicity. The design and synthesis of lanthanide complexes combined with organic ligands have attracted great attention owing to numerous fields of their practical applications in material chemistry and in biosciences [3,4,5,6]. It was found that complexation with lanthanides could enhance the antibacterial [7,8], antifungal [7,9], antioxidant [10], or anticancer activity [11] of free organic ligands. As the potential utility of lanthanides in these areas continues to increase, a study of a lanthanum(III) complex with a 1,2,3-triazole derivative as a bioligand is shown in the present manuscript. The molecule selected as a bioligand for the synthesis of the La(III) complex was the sodium salt of 2-(4-chlorophenyl)-5-(pyrrolidin-1-yl)-2*H*-1,2,3-triazole-4-carboxylate, which is called molecule **2b** according to the notation of Ref. [12], where its synthesis was described (Figure 1).

Numerous lanthanide(III) complexes have been reported to have exhibited pronounced cytotoxic activity on a wide range of tumor cell populations of different origins, with these complexes always showing a significantly higher inhibition potential than the initial ligands and inorganic salts. This class of tumor-inhibiting metal complexes has been shown to be effective against tumors resistant to classical platinum complexes. Their influence on the reactive species (RS) levels generated by several reactive oxygen species (ROS) model systems has also been studied [13] by us, confirming the antioxidant capacity of lanthanide(III) complexes [14].

**Figure 2:**
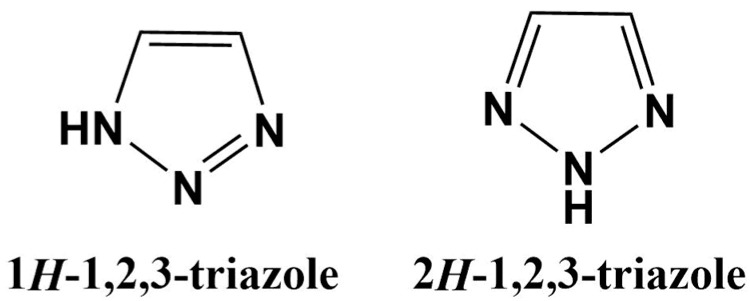
Structure of the 1,2,3-triazole heterocycle.

1,2,3-triazoles (Figure 2) have been extensively studied for their antimicrobial, anticancer, antioxidant, etc., activities [15,16,17,18,19]. This wide range of pharmacological effects is due to their good accessibility; the ease with which their specific structures can be modified with a great variety of substitution patterns; and their photochemical and physicochemical properties, such as a high dipole moment, molecular rigidity, and intermolecular interactions (dipole–dipole, hydrogen bonds, and π-stacking) with biotargets. Therefore, these biologically active compounds are the focus of consideration in several scientific areas, including chemistry, biology, medicine, and physics.

**Figure 3:**
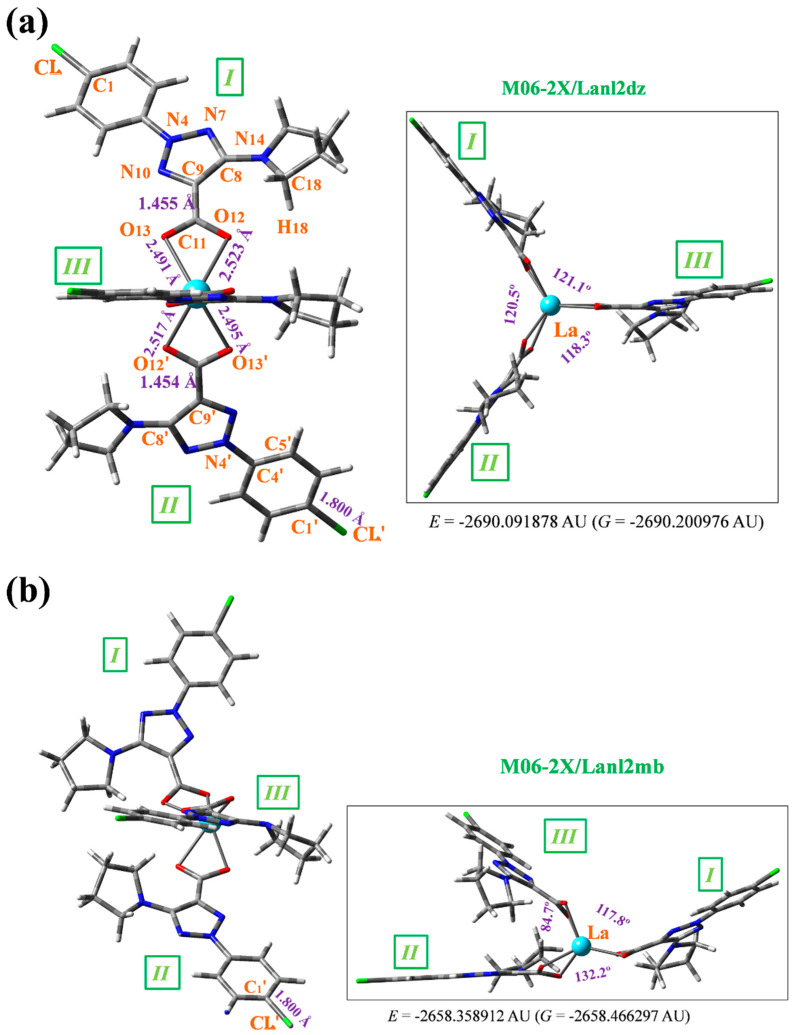
Labeling of the atoms and plot of the optimized La(**2b**’)_3_ structure with **2b**: sodium 2- (4-chlorophenyl)-5-(pyrrolidine-1-yl)-2*H*-1,2,3-triazole-4-carboxylate. Front and lateral view forms were included in the optimized structures at the (**a**) M06-2X/Lanl2dz level and (**b**) M06-2X/Lanl2mb level. Several calculated bond length values of interest are included in the figure, together with the energy values of the system; 1 AU = 2625.5 kJ/mol.

The ligand 2-(4-chlorophenyl)-5-(pyrrolidin-1-yl)-2*H*-1,2,3-triazole-4-carboxylic acid (**1b**) in its anionic form (**2b**) was selected for several reasons: (i) It is a triazole derivative, belonging to a class of molecules widely synthesized and extensively tested nowadays for their anticancer activities [4,11]. (ii) It includes a 1,2,3-triazole ring, which has a remarkable biological potential through different modes of action [13,20]. (iii) It is a novel compound, synthetized by us, bearing a strong-accepting group (the carboxylate) and, in addition, aryl and pyrrolidine groups, providing the structure with liposolubility to facilitate cell membrane permeation. In the complex, La^+3^ ions are coordinated through the carboxylate group (COO^−^) in a three-dimensional system, labeled as La(**2b**’)_3_, as shown in Figure 3. This arrangement finds support in several previously reported lanthanide(III) coordination complexes with carboxylic acid derivatives [14,21].

In the present paper, the identification and the structural and vibrational characterization of the newly synthetized lanthanum(III) coordination complex through theoretical and spectroscopic results were carried out by means of spectral (FT-IR and FT-Raman) and elemental analyses in particular.

The reported work can be regarded as a continuation of our earlier long experience with lanthanide(III) complexes with a number of biologically active ligands. In previous studies, we described the geometry optimization and vibrational characterization of different Ln(III) model systems to suggest the metal–ligand binding mode by using high-level theoretical methods [14,21]. This detailed theoretical approach, along with the experimental data, helped to correctly predict the metal coordination polyhedron of the studied La(III) complex. Due to its poor solubility, we did not succeed in obtaining a single crystal suitable for an X-ray diffraction analysis. Since crystal structure data are not available, theoretical approaches for the determination of the geometrical parameters, vibrational frequencies, hydrogen bond strengths, and the binding mode of the model La(III) complex at a high level of theory are very helpful for extracting reliable structural information. Therefore, we undertook a combined theoretical and experimental vibrational study aiming to determine the binding mode of the ligand and the molecular geometry of its complex. The applied methodology proved to be reliable for the series of lanthanide complexes, and the results are in good agreement with those existing in the literature.

Theoretical and experimental FTIR and Raman spectra of related triazole molecules have been reported [15], although not in such a large complex as presented here or in a detailed form using accurate scaling procedures. Significant differences were observed in the bands of the IR and Raman spectra of the complex compared to those of the free ligand. A comparative vibrational analysis allowed for a good assignment of the functional group vibrations involved in the coordination and helped to explain the ligand vibrational modes, which are sensitive to interactions with metal ions, being in agreement with the literature data and with theoretical predictions.

The impact of the ligand and its complex with La(III) on a variety of RS-generating model systems was assessed—hydroxyl radicals (OH^●^), superoxide (O_2_^●−^), and hypochlorite (OCl^−^)—with some interesting effects having been observed. The potential mechanisms of scavenging activity were additionally elucidated with the help of the 2,2-diphenyl-1-picrylhydrazyl (DPPH^●^) and 2,2′-azino-bis(3-ethylbenzothiazoline-6-sulfonic acid) (ABTS^●+^) stable radical model systems.

## 2. Materials and Methods

### 2.1. Experimental Details

The composition of the newly obtained metal complex was characterized using an elemental analysis. The nature of the La(III) complex was confirmed using IR and Raman spectroscopy. The compounds used for the synthesis of the complexes were from Merck company, p.a.grade: La(NO_3_)_3_·6H_2_O. The sodium salt of **1b** [12] was used as a ligand. The complex was obtained via an interaction between lanthanum(III) inorganic salt and the ligand in an amount equal to a metal:ligand molar ratio of 1:3. The synthesis was carried out by adding an aqueous solution of La(III) nitrate to an aqueous solution of the ligand. The reaction mixture was stirred with an electromagnetic stirrer at room temperature for one hour. As a result of solution mixing, the precipitate of the complex was obtained, which was filtered, washed with water, and dried in a desiccator until constant weight. The obtained complex was very slightly soluble in water, methanol, and ethanol, and it was soluble in dimethyl sulfoxide (DMSO).

The carbon, hydrogen, and nitrogen contents of the obtained complex were determined using an elemental analysis. The content of the metal ion was determined after mineralization. The water content in the complex was determined thermogravimetrically. The obtained elemental analysis data of the new La(III) complex served as a basis for the determination of its empirical formula, and they are presented below.

Solid-state infrared spectra of the complex were recorded in KBr in the 4000–400 cm^−1^ frequency range using an FTIR IFS25 Bruker spectrometer. Raman spectra of the acidic form (**1b**) and the sodium salt (**2b**) of the ligand and its new La(III) complex were recorded with a Dilor microspectrometer (Horiba-Jobin-Yvon, model LabRam) equipped with 1800 grooves/mm holographic grating. The 514.5 nm line of an argon ion laser (Spectra Physics, model 2016) was used for the probes’ excitation. The spectra were collected in backscattering geometry with a confocal Raman microscope equipped with an OlympusLMPlanFL 50× objective. The detection of the Raman signal was carried out with a Peltier-cooled CCD camera. A laser power of 100 mW was used in our measurements.

All UV-VIS measurements were carried out using a Shimadzu UV1601 spectrophotometer. LDCL measurements were performed with an LKB 1251 luminometer (Bioorbit, Turku, Finland) set at 37 °C and connected with an IT-type computer via a serial interface. Three replicate measurements were carried out for each concentration tested. Each measurement represented an individual datapoint. Averages and standard deviations were calculated. Relative changes within the limits of experimental error were not discussed. A one-way ANOVA, followed by a Bonferroni post-test, was used for the statistical verification of the impact of the varying concentrations on the results obtained.

Radical scavenging assays: Preliminary research on the ligand showed its large potential antioxidant properties [22]; these also appear in its lanthanum(III) complex, which has now been analyzed. To conduct the analysis, additional radical-generating model systems (Fenton reaction, potassium superoxide, sodium hypochlorite) were applied to both the ligand and complex, yielding somewhat unexpected and, hence, interesting results.

### 2.2. Materials

*Pro-analysis*-grade materials from SIGMA-ALDRICH (Sigma-Aldrich Chemie GmbH, Taufkirchen, Germany) were used. Solutions were prepared with bi-distilled water and 95% ethanol. The Deoxyribose Degradation Assay involved the following solutions and reagents: 50 mM K-Na-phosphate saline solution (PBS) with pH = 7.45; 6.0 mM water solution of deoxyribose; 3% water solution of trichloroacetic acid (TCA); and 1% water solution of thiobarbituric acid (TBA). The Fenton reaction MTT assay involved the following: 3 mg/mL water solution of 3-(4,5-dimethylthiazol-2-yl)-2,5-diphenyltetrazolium bromide (MTT); Fenton reagent, containing FeCl_2_, H_2_O_2_, and Na_2_-EDTA; and 4 mg/mL water solution of ascorbic acid. The ability to participate in HAT reactions was measured according to well-established protocols [23,24], using a 0.6 mM stock ethanol solution of the 2,2-diphenyl-1-picrylhydrazyl radical (DPPH^●^). The ability of the substances to participate in SET was established using the 2,2′-azino-bis(3-ethylbenzothiazoline-6-sulphonic free radical (ABTS) assay, as described by Erel et al. [25,26]. The following solutions were prepared: 0.4 mM acetate buffer, pH = 5.8, and 0.03 mmol acetate buffer solution, pH = 3.6, with ABTS and H_2_O_2_ in order to produce the stable radical ion ABTS^●+^. The scavenging of superoxide radical ions (O_2_^●−^), derived from potassium superoxide (KO_2_) and hypochlorite (ClO^−^), derived from sodium hypochlorite (NaClO), was assessed with luminol-dependent chemiluminescence (LDCL). 5-Amino-2,3-dihydro-1,4-phthalazinedione (luminol) was dissolved in 0.1 M NaOH and diluted to 1 × 10^−3^ M in PBS of pH 7.45, and the pH was adjusted again to 7.45. A 1 mM KO_2_ solution in dehydrated dimethyl sulfoxide (DMSO) and a 4 × 10^−4^ M aqueous solution of NaOCl were prepared prior to measurements.

### 2.3. Methods

#### 2.3.1. Deoxyribose Degradation Assay

A modified protocol [27] was applied to generate hydroxyl radicals (OH^●^) via water radiolysis [28]. 2-Deoxyribose degradation to MDA-like products was elucidated with the aid of the TBA assay. Every sample contained the following solutions: 0.5 mL 2-deoxyribose, the compound under investigation, and PBS up to 5.0 mL. Controls did not contain the investigated compounds. After 30 min of irradiation with a UV lamp (220–400 nm), 1.0 mL from each 5.0 mL volume was withdrawn. To each of these 1.0 mL volumes, 0.6 mL TCA and 0.6 mL TBA were added, followed by 30 min of cultivation in a water bath at 100 °C. Absorbance was measured at λ = 532 nm. The degree of 2-deoxyribose degradation is presented as the Spectrophotometric Scavenging Index (SPh-SI):

(1) $$SPh-SI=\frac{A_{sample}}{A_{control}}\;\times \;100$$

#### 2.3.2. Fenton Reaction MTT assay

Fenton reaction-generated OH^●^ reduce yellow MTT to purple formazan. Ascorbic acid increases RS production in the model system. Formazan production is registered as an absorbance increase at λ = 578 nm using the kinetic function of the apparatus: 10 s lag time and 600 s measuring time. The sample composition (2.0 mL total volume) is shown in Table 1.

The results are presented as the radical scavenging activity (RSA), calculated with the following formula:

(2) $$RSA,\%=\frac{A_{control}-(A_{sample}-A_{blank})}{A_{control}}\;\times \;100$$

where ΔA_control_ is the relative change in absorbance at 578 nm in the presence of the Fenton reagent but in the absence of the investigated compound. ΔA_sample_ is the relative change in absorbance at 578 nm in the presence of the Fenton reagent together with the investigated compound. ΔA_blank_ is the relative change in absorbance at 578 nm in the absence of the Fenton reagent but in the presence of the investigated compound.

#### 2.3.3. DPPH Assay

The characteristic signal of DPPH^●^ at λ = 517 nm was measured in three types of samples—“blank”, “control”, and “sample” (Table 2).

The total volume of each sample was 2.0 mL. The absorbance decrease was measured using the kinetic function of the apparatus: 10 s lag time and 300 s measuring time. The results are presented as RSA:

(3) $$RSA,\%=\frac{A_{control}-(A_{sample}-A_{blank})}{A_{control}}\;\times \;100$$

#### 2.3.4. ABTS Assay

Two solutions were prepared, as previously described [25]—Reagent 1 (R1) and Reagent 2 (R2). R1 was a Na-acetate buffer with pH = 5.8. R2 contained ABTS in a Na-acetate buffer with pH = 3.8 and H_2_O_2_ to produce the stable ABTS^●+^ radical ion. The total volume of each sample was 1.0 mL. The characteristic signal for ABTS^●+^ was measured at 660 nm. The absorbance decrease was measured using the kinetic function of the apparatus: 10 s lag time and 300 s measuring time. The results are presented as RSA—the same as with the DPPH assay. The sample composition is shown in Table 3.

#### 2.3.5. LDCL in Presence of KO_2_

The 1.0 mL “control” volume included 0.05 mL luminol, 0.05 mL KO_2_, and PBS. The 1.0 mL “sample” volume included 0.05 mL luminol, 0.05 mL KO_2_, the investigated compound at the desired concentration, and PBS. Kinetics were measured with a 2 s delay for a duration of 10 s. Integral intensities were used in the calculations. The results were calculated as the chemiluminometric scavenging index (CL-SI):

(4) $$CL-SI,\%=\frac{I_{sample}}{I_{control}}\times 100$$

The background signal was measured in the absence of KO_2_ and was subtracted from both “control” and “sample”.

#### 2.3.6. LDCL in Presence of NaClO

The 1,0 mL “control” volume included 0.15 mL NaClO, 0.05 mL luminol, and PBS. The 1.0 mL “control” volume included 0.15 mL NaClO, 0.05 mL luminol, the investigated compound at the desired concentration, and PBS. The background signal was measured in the absence of NaClO and was subtracted from both “control” and “sample”. The results are presented as CL-SI, as with the KO_2_ assay.

### 2.4. Computational Details

Density functional methods (DFTs) [29] were only used for the calculations due to the large size of the La(**2b**’)_3_ complex. DFT computations have also provided results for biomolecules, which are quantitatively in good accordance with those obtained at the MP2 level [30,31]. The Minnesota functional M06-2X DFT method was selected because it is the best choice among other meta-generalized gradient functionals to examine dispersion–bound systems [32,33]. Since non-covalent weak interactions were expected in our large system, it was the preferred method. In addition, it also shows broad applicability in chemistry [34,35]. The B3LYP DFT method was also used because it leads to excellent results in IR and Raman vibrational wavenumber calculations [36]. All these methods were implemented in the GAUSSIAN-16 program package [37]. The UNIX version with standard parameters of this package was used on the Brigit super-computer of the University Complutense at Madrid.

For the lanthanide atom, few basis sets are available. The Lanl2dz basis set was chosen because it gave good results in a La(III) complex with a carboxylic acid derivative [14]. Similar to that, the Lanl2mb basis set was also used. Another type is Cep-4g. These are very small basis sets that are available, but they should only be used for compounds with heavy metal atoms, such as the lanthanide atom.

Harmonic wavenumber calculations were carried out at the same level of the corresponding optimization process. All optimized structures showed only positive harmonic vibrations, which indicates a local minima energy. The calculated Raman scattering activities (S_i_) were converted into relative Raman intensities (I_i_) using the relationship derived from the basic theory of Raman scattering [38].

## 3. Results

One of the goals of the present manuscript was to identify and characterize the synthetized La(**2b**’)_3_ coordination complex through a detailed study of the spectroscopic results. In addition, it would be interesting to observe the effect of the La(III) ion on the molecular structure and atomic charge distribution of the **2b** ligand, particularly in the triazole ring. Different basis sets were tested, and the best one was selected.

### 3.1. Synthesis

**Scheme 1:**
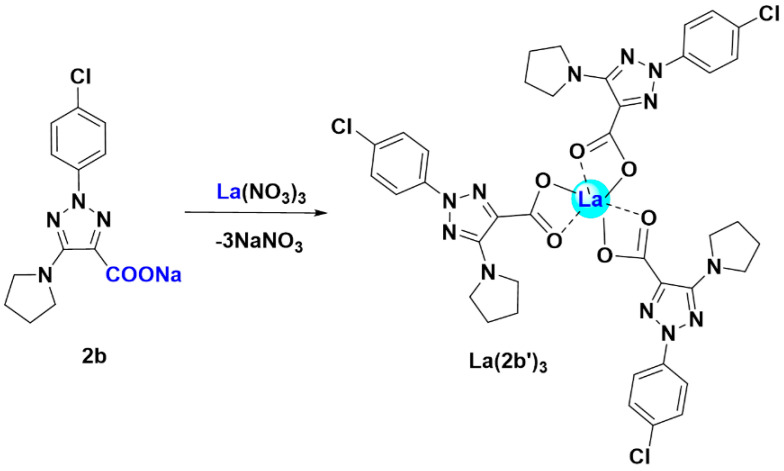
Synthesis of the complex La(**2b**’)_3_.

N-2-aryl-triazoles **1b** and **2b** were prepared according to procedures in the literature. The complex La(**2b**’)_3_ was obtained by treating triazole salt **2b** with La(NO_3_)_3_ at a ratio of 3:1 in a water solution (Scheme 1) with an excellent yield (80%) and characterized using elemental analysis data and IR and Raman spectroscopy.

The elemental analysis of the La(III) complex of 2-(4-chlorophenyl)-5-(pyrrolidin-1-yl)-2*H*-1,2,3-triazole-4-carboxylic acid: (% calculated/found): La(**2b**’)_3._2H_2_O: C: 44.66/45.05; H: 3.82/3.69; N: 16.03/16.38; H_2_O: 3.43/3.87; La: 13.26/13.05, where **2b**’= C_13_H_12_N_4_O_2_Cl^-^.

### 3.2. Molecular Structure of the Complex

Since lanthanide metals coordinate more preferably with oxygen atoms than with nitrogen atoms [14], the starting optimized structure was that with the lanthanum(III) ion coordinated with three **2b** ligands through the carboxylate group (COO^−^) (Figure 3).

Two views of the optimized structure at the M06-2X/lanl2dz level are shown in Figure 3a, with an almost symmetrical arrangement. Via the rotation of the carboxylate group around the C_9_-C_11_ bond, another conformer can be obtained, but it is slightly less stable than that plotted in this figure. The notation used for labeling the atoms is in accordance with the one previously reported for this very same **2b** ligand [12]. A few bond length values are also included in this figure. In the bottom of it, the total energy (E) value is shown, which includes the ZPE (zero-point vibrational energy) correction and the Gibbs energy (G).

The intramolecular H-bond between the carbonyl oxygen O_12_ and the pyrrolidine hydrogen H_18_ is greatly weakened by M06-2X (2.443 Å) in the La-(**2b**’)_3_ complex due to La-O_12_ bond formation, but it is present in the ligand isolated by MP2 (2.034 Å) [39].

The average calculated value of the La-O bond length (2.5 Å) agrees well with the experimental value of the odd diacids, and it is specific to the monometallic complexes of lanthanides [14,40]. An increase in this value has been related to a closing of the OCO angle [41]. The calculated bond angle of C=O_12_-La (94.0°) is slightly different to the C=O_13_-La (95.7°) angle, which indicates that the C-O-La bond is not fully linear due to the slightly higher negative charge of O_13_ (−1.266e) compared to that of O_12_ (−1.250e). A higher negative charge on O_13_ leads to a shortening of the O_13_-La bond length (2.491 Å) vs. O_12_-La (2.523 Å) and, therefore, an opening of the C=O_13_-La angle. This very slight asymmetry is also observed in the O_12_-La-O′_13_ angle (103.0°), which is slightly more open than the O_12_-La-O′_13_ angle (102.0°). These features also lead to the rotation of the ligands, and, thus, the torsional angle C_11_-O_12_···O′_12_-C′_11_ (13.2°) is quite different from the C_9_-C_11_···C′_11_-C′_9_ angle (−3.2°), as well as the (C_11_···La·· C′_11_) angle (121.1°), compared to (C′_11_···La···C′_11_) (118.3°).

In this optimized structure, at the M06-2X/lanl2dz level, the aryl ring is fully planar and almost coplanar with the triazole ring, with a C_5_-C_4_-N_4_-N_10_ torsional angle of 0.8°, although it appears slightly rotated (−2.3°) by MP2. The triazole ring is also planar, with values of the torsional angles lower than 1°. The pyrrolidine ring, however, is noticeably out-of-plane with a value of the torsional angle N_14_-C_15_-C_16_-C_17_ of −32.2°, in accordance with that found in a cyclopyrrol ring. This pyrrolidine ring is also non-coplanar with the triazole ring plane with a value of the torsional angle C_9_-C_8_-N_14_-C_18_ of −18.3°, although it is 32.0° with MP2, indicating a failure in this angle calculation when using the M06-2X method with lower values.

The carbon C_11_ atom of the carboxylate group appears to be slightly titled out of the triazole ring plane and remarkably rotated, but this rotation is larger with O_13_ than with O_12_, with a value of the torsional angle N_10_-C_9_-C=O_13_ of −11.2° vs. C_8_-C_9_-C=O_12_ of −7.5°. For the binding to the lanthanide ion, the OCO angle closes up to 117.4° vs. 131.3° with MP2 in the free ligand, and the CO bond lengths are noticeably lengthened. This large flexibility of the carboxylic oxygens facilitates bonding to the lanthanum(III) ion. The lengthening of the CO bonds leads to a shortening of the C_9_-C_11_ bond length, which noticeably reduces the double-bond character of the N_7_=C_8_ and C_9_=N_10_ bonds of the triazole ring, and, consequently, the N_4_-N_10_ and C_8_-N_14_ bonds are shortened. This feature slightly modifies the bond angles of the triazole ring with lanthanide binding.

The optimized structure at the M06-2X/lanl2mb level appears to be deformed (Figure 3b), perhaps due to its smaller basis set. However, this more planar arrangement can probably be more expected to be present in the solid-state sample due to the crystal packing forces compressing the structure. It is noted that the intramolecular O_12_···H_18_ H-bond only appears in ligand I, and it is very weak in ligands II and III. The C-N and N-N bond lengths are noticeably larger and the La-O bonds are noticeably shorter than with M06-2X/lanl2dz. Because of the structural deformation, the C=O-La and O_12_-La-O′_13_ angles are closer and the O_12_-La-O′_13_ angle is larger than with the lanl2dz basis set. The pyrrolidine and carboxylate groups are also more rotated related to the triazole ring, and the arrangement of the ligands differ remarkably, with a (C′_11_···La···C″_11_) angle of 84.7° vs. 118.3° with the lanl2mb basis set and a torsional angle (C_11_-O_12_···O′_12_-C′_11_) of −169.5° vs. 13.2° with the lanl2mb basis set. However, the value of the OCO angle remains almost the same at both levels.

Due to the optimized structure of the B3LYP/Cep-4g appearing largely deformed, it is included in Figure S1 (Supplementary Material). The low basis set used contributes to this feature, as well as the fact that the B3LYP method does not consider long-range interactions that stabilize the system to a symmetric arrangement, as shown in Figure 3a. The optimized values at this level have noticeably longer bond lengths than with M06-2X and MP2. With longer bond lengths, the triazole substituents are more twisted, especially the pyrrolidine ring, which can interact with other ligands.

### 3.3. Atomic Charges

The lanthanide ion has the highest positive charge, which is 3.164e with M06-2X/Lanl2dz and 2.884e with B3LYP/Cep-4G. Uncommonly, it is rather small (2.066e) with M06-2X/Lanl2mb. As expected, the highest negative charge corresponds to the oxygen atoms, with the charge on O_13_ being slightly higher in the negative value than that on O_12_, and in contrast to that calculated in the free **2b** molecule with MP2. These oxygens appear as the most reactive, and, therefore, they are bidentate-coordinated with the lanthanide ion. They are expected to participate in the biological activity of these molecules. The negative charge on the nitrogen atoms N_4_ and N_14_ is lower, but it is expected that they can also contribute to the biological activity of this compound. By contrast, a high positive charge appears in the carbon atoms C_8_ and C_11_ because they are bonded to large negative atoms.

### 3.4. Vibrational Analysis

For the identification and characterization of the synthesized La(**2b**’)_3_ coordination complex, a detailed analysis of the calculated and experimental spectra was carried out. Low-lying molecular vibrations have been studied by different authors [42,43,44,45], and their presence indicates a high flexibility in the molecular structure that may be caused by various factors. Moreover, in our La(**2b**’)_3_ complex, the normal vibrational modes that include the lanthanide ion mainly appear below 200 cm^−1^. Thus, these kinds of vibrations may play an important role in some biological functions, such as the transcription and replication of the double DNA helix, specific interactions with proteins and drugs, and gene expression [45]. Our ligand molecule **2b** possesses high conformational flexibility, since it was characterized by ten vibrational modes below 200 cm^−1^. These modes may also be important for local recognition in the protein cavity. Moreover, the pattern of individual vibrations for every ligand also reflects the details of its conformation. Taking into account the high deformability of our molecule **2b**, these low frequencies should be very sensitive to changes in the local environment, such as hydration and interactions with other biomolecules.

**Figure 4:**
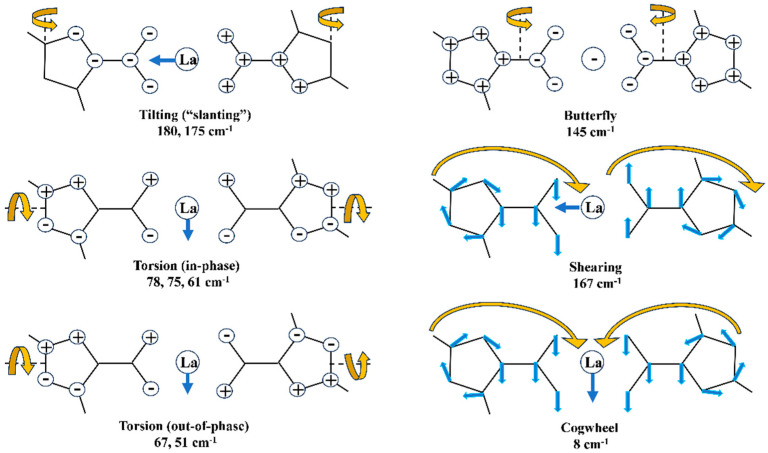
Low-frequency vibrational modes characterized in the calculated spectrum at the M06-2X/Lanl2dz level. The ⊕ and ⊝ symbols correspond to out-of-plane motions in one direction or in the opposite direction.

Additionally, the spectra in the frequency region under 200 cm^−1^ are quite interesting, since they offer information about metal–ligand vibrations. Thus, in the present work, a theoretical analysis of the low-lying vibrations of our La(**2b**’)_3_ complex with frequencies below 200 cm^−1^ was carried out. Because the large number of vibrational modes below 200 cm^−1^ in our complex, 42 in total, only the 6 most characteristic ones were considered and plotted in schematic form in Figure 4. For simplicity and clarity, only the carboxylic and triazole ring vibrations of two of the three ligands are included in this figure. The larger number of these vibrations and their shape, compared to those of the **2b** ligand, agree well with a noticeable increase in the La(**2b**’)_3_ complex flexibility.

The first detail observed in these vibrations is that the lanthanide ion motions do not follow the bond length lines with the carboxylic oxygen atoms. It appears that its motion depends on the modulus and direction of the displacement vectors of the surrounding oxygen atoms. The second detail observed is that most of the vibrational modes follow the displacement vectors calculated in the dimer form of benzoic acid (BA) [46] and in agreement with those found using far infrared [47]. The lanthanide ion does not appear to affect the displacement vectors of the carboxylic group.

As expected in the low-frequency range, in some atoms, the in-plane and out-of-plane characteristics of the displacement vectors are mixed, which makes it difficult to assign them. Figure 4 shows only the most characteristic modes that can be well related to those corresponding to the BA molecule and in which the lanthanide ion has a noticeable large displacement vector. In the remaining vibrational modes, the motions of other groups remarkably prevail over those of the carboxylic group and the lanthanide ion, and, therefore, they are not included in Figure 4. There are also modes that are difficult to characterize. An analysis of the shape of the selected vibrations reveals that most of the modes involve a strong deformation of the complex, and the motion of the whole structure seems, for example, to be a γ (butterfly) and a γ (tilting “slanting”).

### 3.5. Radical Scavenging Assays

It has been reported [22] that the sodium salt of the ligand (**2b**), together with its non-ionic, conjugate acid (**1b**), suppresses in vitro 2-deoxyribose degradation at a concentration of 3 × 10^−5^ M or higher. The scavenging of DPPH^●^ was not observed with **2b**, and a mild effect was observed with 1 × 10^−4^ M **1b** (RSA = 10 ± 2%), decreasing in a concentration-dependent manner to practically zero at 1 × 10^−5^ M. Both compounds scavenge ABTS^●+^ at 1 × 10^−4^ M (RSA = 17 ± 1% with **2b** and RSA= 13 ± 2% with **1b**); again, the effect drops to zero at 1 × 10^−5^ M. These results are presented together with the new data derived from the equivalent experiments on the complex La(**2b**’)_3_.

#### 3.5.1. Impact of **1b**, **2b**, and La(**2b**’)_3_ on 2-Deoxyribose Degradation

**Figure 5:**
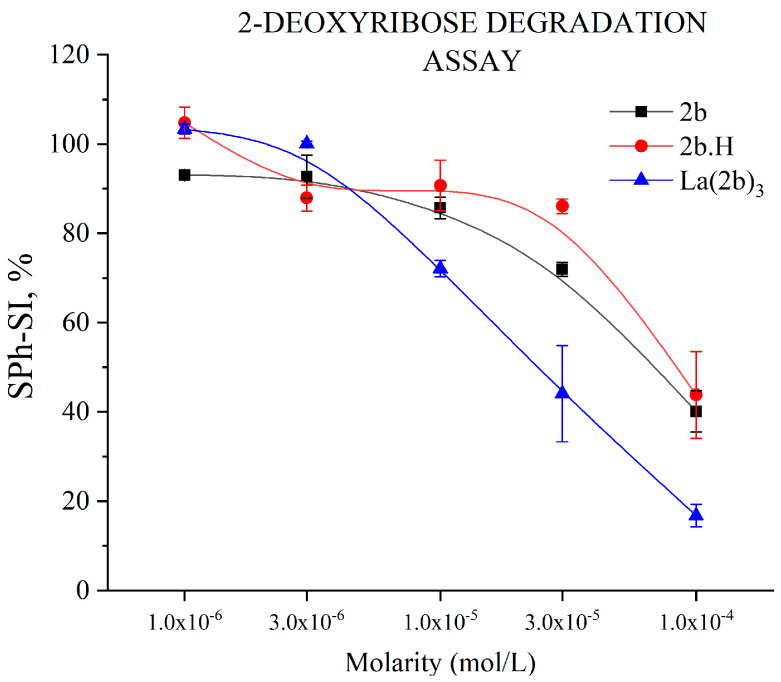
Impact of **1b**, **2b**, and La(**2b**’)_3_ on 2-deoxyribose degradation, resulting from UV−induced water radiolysis. Data = Mean ± StDev, *p* < 0.05, N = 3. Lower results mean higher scavenging activity.

The effects of **2b** and La(**2b**’)_3_ compounds on the UV-induced degradation of 2-deoxyribose are presented in Figure 5. They were investigated at concentrations between 1 × 10^−6^ M and 1 × 10^−4^ M. A statistically significant concentration-dependent effect with respect to the scavenging of OH^●^ was observed with these three compounds. Unlike **2b** and **1b**, the complex manifests significant scavenging activity at a lower concentration (SPh-SI = 72 ± 2% at 1 × 10^−5^ M). At concentrations above 3 × 10^−6^ M, the scavenging activity of the complex is not different than the scavenging activity of **2b** at a three times higher concentration. Each La(III) ion coordinates three **2b** ligands. This feature may be a clue that all three ligands in the complex participate equally in the scavenging process.

#### 3.5.2. Impact of **1b**, **2b**, and La(**2b**’)_3_ on a Model System Containing the Stable Radical DPPH^●^

**Figure 6:**
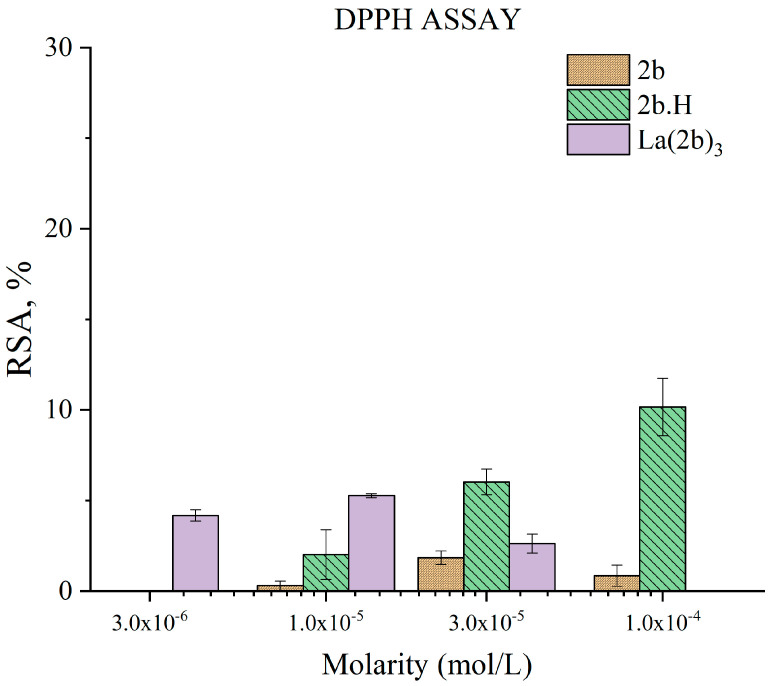
Impact of **2b**, **1b**, and La(**2b**’)_3_ on DPPH^●^. Data= Mean ± StDev, *p* < 0.05, N = 3. Higher result means higher scavenging activity.

The ability of the tested compounds to exchange hydrogen is presented in Figure 6. Previous research showed that **2b** practically does not interact with DPPH^●^, while its conjugate acid **1b** interacts only weakly at 1 × 10^−4^ M (RSA = 10 ± 2%), with the effect decreasing in a concentration-dependent manner to zero at 1 × 10^−5^ M. For this reason, lower concentrations were not studied. The La(III) complex was investigated at three times lower concentrations, manifesting very mild activity within the tested concentration range. An additional concentration of 1·10^−6^ M yielded a result of RSA = 0.5 ± 0.4%. The low activity toward DPPH^●^ means that all three compounds tend to scavenge it via HAT only weakly. One has to consider that DPPH^●^ has a large molecule and that steric hindrances may play a part in the observed low activity. The model system with 2-deoxyribose produces OH^●^ that are known to be scavenged via HAT reactions [48]. Unlike DPPH^●^, OH^●^ are small in size and very mobile. La(**2b**’)_3_ and **2b** demonstrate a clear scavenging effect toward OH^●^, generated by water radiolysis at concentrations higher than 1 × 10^−6^ M. The mild, concentration-dependent DPPH scavenging activity of **1b** may be due to the presence of an active carboxyl hydrogen atom in its molecule. La(**2b**’)_3_ is more active than three times higher concentrations of **2b**. That may be due to the impact of the La(III) ion on the electron density distribution within the coordinated ligands, thus yielding active hydrogen atoms, increasing interactions with DPPH^●^.

#### 3.5.3. Impact of **2b**, **1b**, and La(**2b**’)_3_ on a Model System Containing the Stable Radical ABTS^●+^

**Figure 7:**
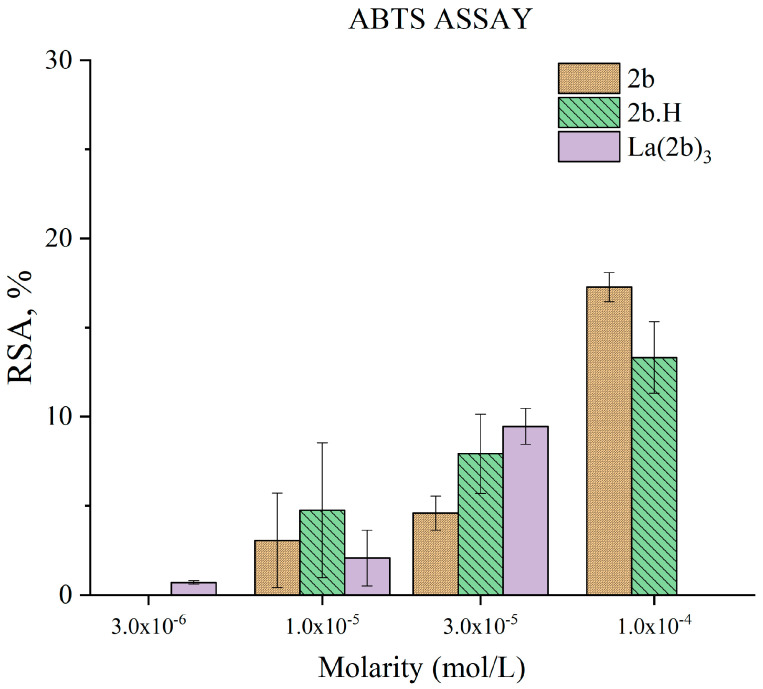
Impact of **2b**, **1b**, and La(**2b**’)_3_ on ABTS^●+^. Data= Mean±StDev, *p* < 0.05, N = 3. Higher result means higher scavenging activity.

The ability of the tested compounds to participate in SET reactions with ABTS^●+^ is presented in Figure 7. At the highest tested concentration of 1 × 10^−4^ M, **2b** and **1b** had a mild effect on this model system, decreasing to practically statistically zero at 1 × 10^−5^ M. For that reason, lower concentrations were not tested. La(**2b**’)_3_ was investigated at three times lower concentrations. Its activity at 3 × 10^−5^ M (RSA = 9 ± 1%) was greater than that of **2b** at the same concentration (RSA = 5 ± 1%). However, 1 × 10^−4^ M **2b** (three times greater concentration) was more active (RSA = 17.3 ± 0.8%) than 3 × 10^−5^ M La(**2b**’)_3_. A similar, statistically significant observation was noted when comparing 1 × 10^−5^ M La(**2b**’)_3_ (RSA = 2.1 ± 1.6%) with 3 × 10^−5^ M **2b** (RSA = 5 ± 1%). At concentrations of 1 × 10^−5^ M, the ligand and the complex did not demonstrate significant electron exchange with ABTS^●+^. La(**2b**’)_3_ tended to be less active than three times more concentrated **2b**. That may be due to the fact that the active sites for electron exchange in the ligand might be sterically hindered after complexation with La(III) occurs. Such a complexation may also cause electron density redistribution due to the presence of the metal coordination center.

#### 3.5.4. Impact of **2b** and La(**2b**’)_3_ on MTT-Formazan Transformation via Fenton Reaction-Derived Hydroxyl Radicals

The Fenton reaction is a well-known chemical process with a clear clinical significance [49]. “Free” transition metal ions within living organisms create the conditions for its fast progress that yields highly reactive hydroxyl radicals. OH^●^ tend to attack molecular sites bearing conjugated double bonds, causing a free radical chain reaction, molecular fragmentation, lipid peroxidation, and MDA formation. Antioxidants can stop the aforementioned processes, thus preventing damage to biomolecules and possible pathogenesis.

**Figure 8:**
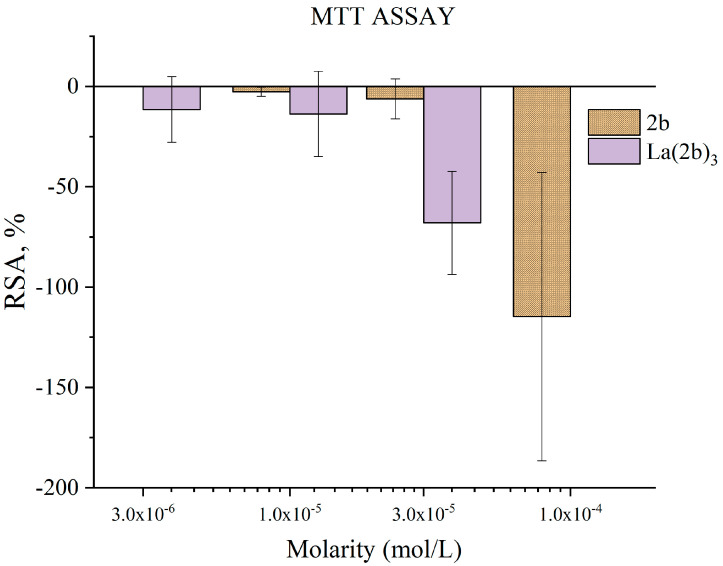
Impact of **2b** and La(**2b**’)_3_ on MTT-Formazan transformation by Fenton reaction-generated OH^●^. Data = Mean ± StDev, *p* < 0.05, N = 3. Higher result means higher scavenging activity.

The water radiolysis model system can serve as a good demonstration of the interaction between OH^●^ and a potential antioxidant. However, the Fenton reaction is a significant biochemical process that takes place under physiological conditions in living organisms. Therefore, what would be expected is similarity between the results of these model systems. That is what makes the results presented in Figure 8 even more interesting.

The complex was tested at concentrations between 3 × 10^−6^ M and 3 × 10^−5^ M. The ligand **2b** was tested at three times greater molarities. In the presence of both compounds, the behavior of this model system was extremely unstable. Experiments were repeated twice on different days to lower the probability of human error or equipment failure as much as possible. Based on the observations made, both compounds increase formazan production; i.e., they increase RS formation, acting as prooxidants. At concentrations up to 3 × 10^−5^ M, the ligand **2b** manifests no statistically significant impact on absorbance at 578 nm or, hence, on RSA. At 1 × 10^−4^ M, the effect is strong and statistically significant (RSA = 115 ± 72%). At a three times lower molarity, La(**2b**’)_3_ also seems to act as a prooxidant, though to a smaller degree (RSA = 68 ± 16%). At lower concentrations, the effect is statistically zero. These results are unexpected for the authors—in the water radiolysis system, both the ligand and the complex act as potent hydroxyl radical scavengers, while, at the same time, MTT-formazan transformation, caused by Fenton reaction-generated OH^●^, seems to be potentiated by these same substances.

#### 3.5.5. Impact of **2b** and La(**2b**’)_3_ on LDCL in Presence of KO_2_

**Figure 9:**
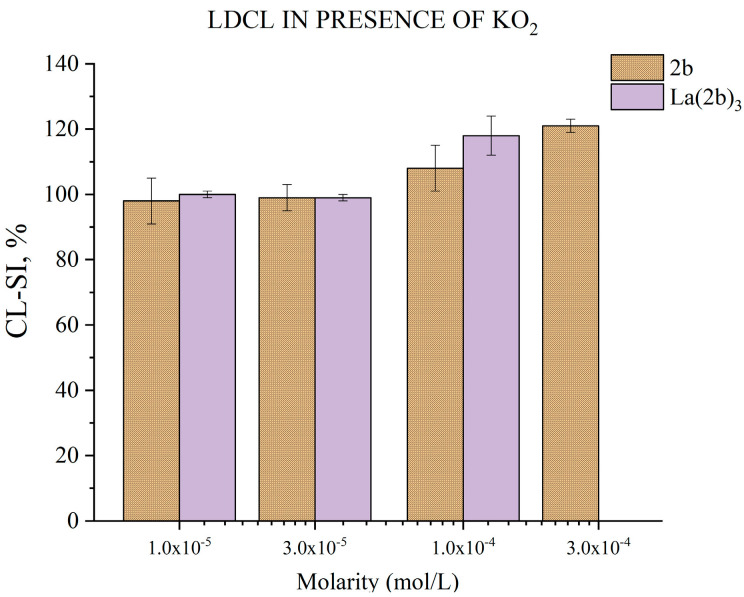
Impact of **2b** and La(**2b**’)_3_ on LDCL from KO_2_. Data = Mean ± StDev, *p* < 0.05, N = 3. Lower result means higher scavenging activity.

The superoxide radical ion is a type of RS that normally occurs in the human body, produced by a one-electron reduction of oxygen. As a by-product of oxygen metabolism and various dedicated enzymes, it also plays a role in the defense against pathogens and in a variety of cell signaling pathways [50,51]. The ability of the ligand **2b** and Ln(**2b**’)_3_ to scavenge KO_2_-derived superoxide is presented in Figure 9.

At concentrations of 3 × 10^−3^ M and below, both compounds are inactive. At 1 × 10^−4^ M, the ligand increases LDCL slightly (CL-SI = 108 ± 7%). The effect becomes more evident at 3·10^−4^ M (CL-SI= 121 ± 2%)—evidence of slight prooxidant action. At 1 × 10^−4^ M, La(**2b**’)_3_ has CL-SI = 118 ± 6%. The limited solubility of both compounds does not allow for the testing of higher molarities. In terms of in vitro KO_2_ superoxide scavenging, both the ligand and its complex could be viewed as relatively inert.

#### 3.5.6. Impact of **2b** and La(**2b**’)_3_ on LDCL in Presence of NaClO

**Figure 10:**
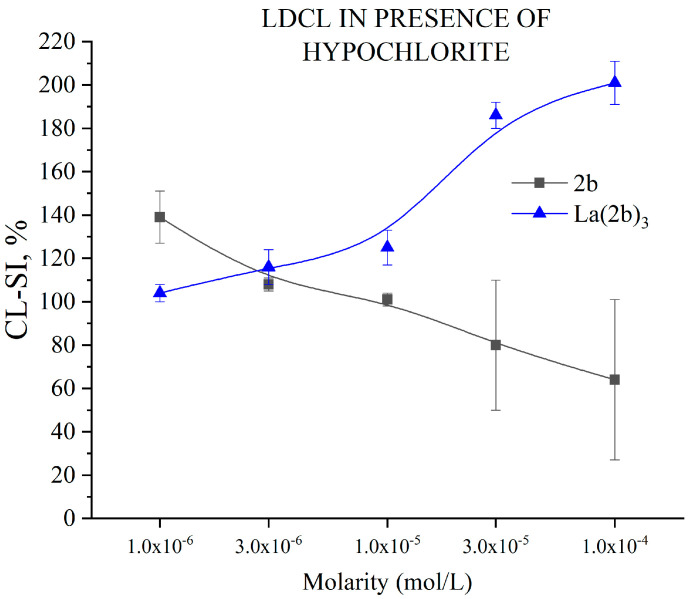
Impact of **2b** and La(**2b**’)_3_ on LDCL from hypochlorite. Data = Mean ± StDev, *p* < 0.05, N = 3. Lower result means higher scavenging activity.

Hypochlorous acid is naturally produced in the human body as an important component of immune defense [52]. It is produced by neutrophils during the respiratory burst—the enzyme myeloperoxidase uses H_2_O_2_ and chloride ions as substrates to synthesize this highly toxic species, a strong oxidant that damages a variety of molecular targets, acting as a bactericide. Its high chemical reactivity and non-specific action are the reason why HClO is associated with a variety of human pathologies [53]. The ability of the ligand **2b** and Ln(**2b**’)_3_ to scavenge in vitro NaClO-derived superoxide is presented in Figure 10.

When it comes to in vitro interactions with hypochlorite, the divergent trends of behavior are quite stark. At the lowest tested concentration, the ligand **2b** increases LDCL (CL-SI = 139 ± 12%) quite significantly compared to the control. As molarity rises, CL-SI decreases in a concentration-dependent manner down to 64 ± 40% at 1 × 10^−4^ M. The opposite is observed with Ln(**2b**’)_3_. At 1 × 10^−6^ M, it does not significantly impact LDCL (CL-SI = 104 ± 4%). At the next higher concentration (3 × 10^−6^ M), CL-SI increases and is statistically the same as that of **2b**. That increase continues right up to 201 ± 10% at 1 × 10^−4^ M. A previous study [54], using the same model system with another La(III) complex, demonstrated a clear correlation between the behavior of both the ligand and complex. In this case, the coordination of **2b** with La(III) seems to show some kind of drastic change in behavior.

## 4. Conclusions

A new lanthanum(III) complex of 2-aryl-1,2,3-triazole was synthesized and characterized using an elemental analysis, and IR and Raman spectroscopy. The analysis was carried out in detail to confirm the molecular arrangement of the ligands in the newly synthetized complex. The most important findings are the following:

- A structural study of the lanthanum(III) complex was carried out at three DFT levels. The starting optimized structure was that with the La(III) ion coordinated with three **2b** ligands through the carboxylate group. The optimized structure at the M06-2X/lanl2dz level showed an almost symmetric arrangement. By rotation around the C_9_-C_11_ bond length, another conformer could be obtained, but it was less stable.
- Global chemical reactivity descriptors were calculated in the La(**2b**’)_3_ complex. The low energy gap calculated indicated a large chemical reactivity and small excitation energies to the manifold of excited states.
- The coordination of the **2b** ligand with the La(III) ion noticeably changed its IR and Raman spectra, which seemed different to those obtained with **2b** alone.
- The main low-lying molecular vibrations in the La(**2b**’)_3_ complex were characterized. Their number indicated a high flexibility in its molecular structure.
- The complex showed moderate HAT activity with the stable DPPH^●^ radical, but the activity of ligand **2b** in this model system was much lower, that is, almost zero. This lack of activity may be due to steric factors that hinder hydrogen transfer between the bulky DPPH^●^ and the possible hydrogen-donating active sites in the **2b** and La(**2b**’)_3_ molecules. When interacting with the small and mobile OH^●^, generated by UV-induced water radiolysis, both the ligand and complex behaved as scavengers (3 × 10^−6^ M or higher), with the latter being the more potent at equimolar concentrations.
- The ligand and the complex participated in SET with the ABTS^●+^ radical ion only moderately, in a concentration-dependent manner. Steric factors also seemed to be at play here, as the complex appeared to be less active than the ligand at three times the concentration.
- Both compounds appeared to have little impact on KO_2_-derived superoxide. Only at the highest tested concentrations, 3 × 10^−4^ M for **2b** and 1 × 10^−4^ M for La(**2b**’)_3_, did they seem to show a slight significant prooxidant effect.
- Though **2b** and La(**2b**’)_3_ scavenged OH^●^, a different model system (Fenton reaction), generating the same RS yielded completely opposite results. At low concentrations, both compounds seemed to be inactive. Increasing the molarity to 3 × 10^−5^ M and 1 × 10^−4^ M for La(**2b**’)_3_ and **2b**, respectively, yielded results demonstrating that these compounds behave as prooxidants in this model system. As possible causes of this behavior, it is proposed that the ligand interacts with one or more components of the system rather than with the OH^●^ generated by it.
- The La(III) ion dramatically changed the behavior of **2b** toward hypochlorite once coordination took place. The higher the concentration, the better the hypochlorite scavenger **2b** seemed to be. The behavior of its La(III) complex was the opposite, manifesting itself as a potent prooxidant at the highest concentration tested.

The structural and spectroscopic characterization of this new triazole derivative La(III) complex with possible anticancer and antioxidant activities could facilitate the characterization of new complexes with other lanthanides. Its in vitro behavior toward a wide range of RS-generating model systems certainly revealed some interesting possibilities for potential therapeutic applications: compounds that act as hydroxyl scavengers in certain conditions, but as potential prooxidants in others. The La(III) ion is known to be able to compete with iron, displacing it from binding sites in transport proteins [55] and iron-dependent enzymes. Thus, both enzyme inhibition and “free” iron release take place simultaneously [56]. Free iron produces RS through the Fenton reaction [57]. A La(III)-bearing complex that can impair iron-dependent physiological processes, release “free” iron, and allow the Fenton reaction to take place in addition to enhancing it may very well be a promising avenue for further investigation in the oncology field. LDCL demonstrates that, while **2b** may act as a hypochlorite scavenger, La(**2b**’)_3_ may well be a hypochlorite “enhancer”, bearing a coordination center with intrinsic antibacterial activity—another possible antimicrobial application that may well be worth investigating.

## Figures and Tables

**Table 1:** Fenton reaction MTT assay sample composition.

	Control	Sample	Blank
Tested compound	no	200 μL	200 μL
MTT	200 μL	200 μL	200 μL
Fe^2+^/H_2_O_2_/Na_2_-EDTA	100 μL	100 μL	no
Ascorbic acid	100 μL	100 μL	no
Bi-distilled water	up to 2.0 mL	дo 2.0 mL	дo 2.0 mL

**Table 2:** DPPH assay sample composition.

	Blank	Control	Sample
Tested compound	200 μL	no	200 μL
DPPH	no	1800 μL	1800 μL
Ethanol	1800 μL	no	no
Bi-distilled water	no	200 μL	no

**Table 3:** ABTS assay sample composition.

	Blank	Control	Sample
Tested compound	100 μL	no	100 μL
R1	860 μL	860 μL	860 μL
R2	no	40 μL	40 μL
Bi-distilled water	40 μL	100 μL	no

## Data Availability

Not applicable.

## Supplementary Materials

The following supporting information can be downloaded at https://www.mdpi.com/article/10.3390/antiox12101872/s1, Figure S1: Labeling of the atoms and plot of the optimized La(**2b**)3 structure with **2b**: sodium 2-(4-chlorophenyl)-5-(pyrrolidineidin-1-yl)-2H-1,2,3-triazole-4-carboxylate at the B3LYP/Cep-4g level.
