# Supplementary material for: Structural Study of a La(III) Complex of a 1,2,3-Triazole Ligand with Antioxidant Activity
Source: Antioxidants (Basel). 2023 Oct 17;12(10):1872. doi: 10.3390/antiox12101872 (PMC10604163; doi:10.3390/antiox12101872)
Supplement: Supplementary file 1 [file antioxidants-12-01872-s001.zip › antioxidants-2649060-supplementary.pdf]

SUPPLEMENTARY MATERIAL

1,2,3-triazole-Lanthanide complex with anticancer activity: structural and spectroscopy

M. Alcolea Palafox[a*], Nataliya P. Belskaya[b], Lozan Todorov[c], Irena P. Kostova[c]

[a] *Departamento de Química Física, Facultad de Ciencias Químicas, Universidad Complutense, Madrid-28040, Spain* (alcolea@ucm.es)
[b] *Department of Technology for Organic Synthesis, Ural Federal University, 19 Mira Str., Yekaterinburg 620012, Russia; n.p.belskaya@urfu.ru*
[c]*Department of Chemistry, Faculty of Pharmacy, Medical University – Sofia, 2 Dunav Str., Sofia, Bulgaria*

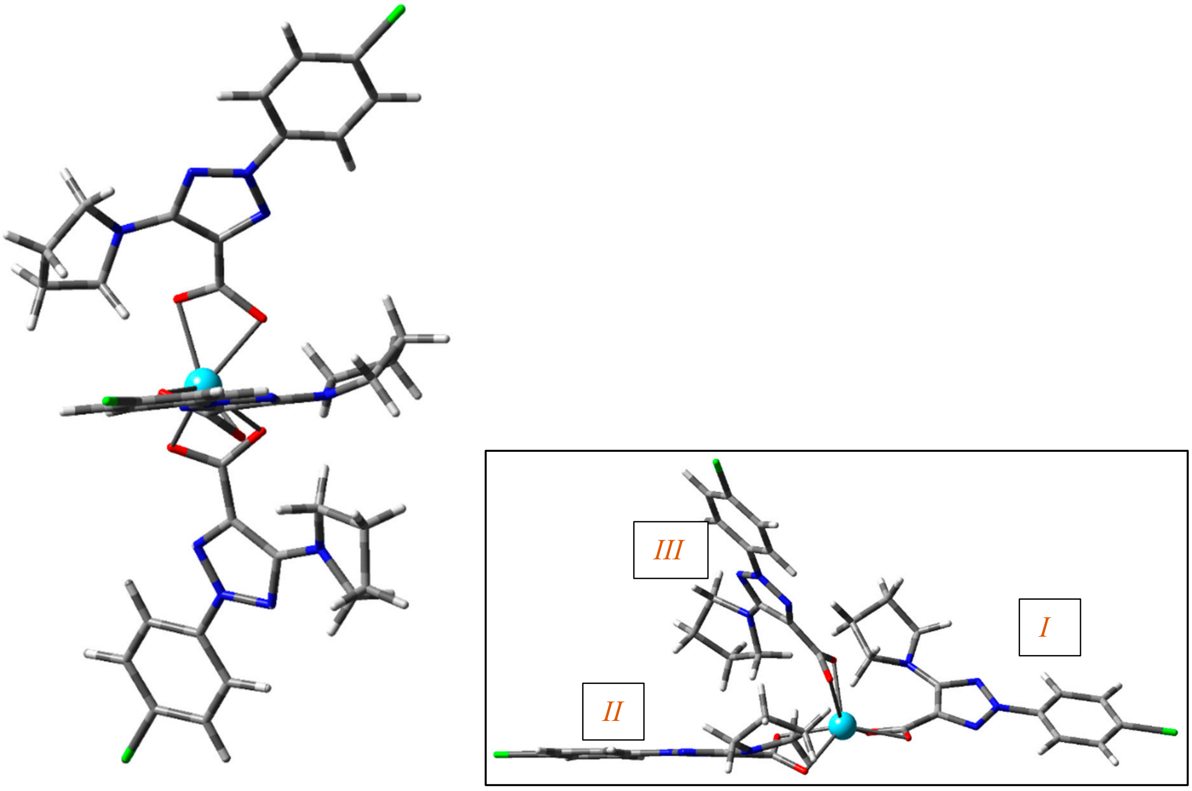

E = -528.387053 AU (*G* = -528.502017 AU)

Figure S1. Labeling of the atoms and plot of the optimized La(**2b**)$_3$ structure with **2b**: sodium 2-(4-chlorophenyl)-5-(pyrrolidineidin-1-yl)-2*H*-1,2,3-triazole-4-carboxylate at the B3LYP/Cep-4g level.
